# Diagnosis and treatment of erectile dysfunction– a practical update

**Published:** 2009-11-25

**Authors:** C Persu, V Cauni, S Gutue, ES Albu, V Jinga, P Geavlete

**Affiliations:** * Urology Department, ‘Saint John’ Emergency Clinical Hospital, BucharestRomania; ** Department of Urology,‘Th. Burghele’ Clinical Hospital, BucharestRomania; *** Department of Obstetrics and Gynecology, University Emergency Hospital, BucharestRomania

**Keywords:** Erectile dysfunction (ED), PDE–5 inhibiter, penile prosthesis

## Abstract

During the last decades, erectile dysfunction was considered a direct consequence of aging and, although of a great concern for the affected patient, little was available to evaluate and treat this problem. If aging could not be invoked in all cases, than the psychogenic etiology was the only explanation. Over the coming years, a better understanding of the physiology involved in the penile process of tumescence and detumescence has allowed for better approach of each disease asociated with erectile dysfunction so that adequate treatment modalities can be applied to the pacient. As we all know, every pacient is a particular case. The development of modern PDE–5 inhibiters, along with other more or less invasive therapies, puts a new light on the medical approach of ED.

## Etiology of ED

Erectile dysfunction (ED), defined today as the constant inability to attain and mantain an erect penis with sufficient rigidity for sexual intercourse, has become an increasingly common presenting complaint and is estimated to 3% of young men with ages between 15 and 25 years old and 7% of those with age between 25 and 45 years old suffer from ED, in Romania. This percentage increases up to almost 22% of men with ages between 45 and 55 years old [1]. These numbers are referring to secondary ED, which implies the loss of previously normal potency. Also, it has been estimated that impotence affects 140 million men worldwide.(Table 1)

**Table 1:** Differentiation between Psychogenic and Organic ED

Characteristic	Organic	Psychogenic
Onset	Gradual	Acute
Circumstances	Global	Situational
Course	Constant	Varying
Noncoital erection	Poor	Rigid
Psychosexual problem	Secondary	Long history
Partner problem	Secondary	At onset
Anxiety and fear	Secondary	Primary

*Psychological causes* (e.g. depression, anxiety and stress involving the workplace) used to be considered some time ago as the most common reason for ED [1], especially in young men, but are now thought to be the primary factor in only a few cases. However,secondary psychological problems are expected in all cases associated with ED.

Despite initial beliefs considering that the vast majority of ED cases had a psychogenic etiology, modern day's medicine proved that Organic causes are responsible for about 60% to 90% of all causes of ED. Most frequent pathologies involved are:

- *Vasculogenic*. The most common single cause, due to low blood inflow (e.g. large vessel atherosclerosis). The incidence of ED in atheromatous aortoiliac and peripheral vascular disease is about 50%. On the other side, increased outflow, also known as venous leak or venogenic ED, may be responsible. The venous outflow regulatory mechanism depends on the completeness of trabecular smooth muscle relaxation and the expandability of the erectile tissue, defined as the ability to achieve maximal corporal volumes at low intracavernosal pressures. Arteriogenic and venogenic ED can also coexist in the same patient.
- *Diabetes mellitus* is a common cause of organic ED, up to 75% of diabetic patients accusing poor erections. It is hypothesized that cavernosal artery insufficiency, corporal venoocclusive dysfunction, and/or autonomic neuropathy are the major organic pathophysiologic mechanisms leading to persistent erectile impairment in men with diabetes mellitus.
- *Endocrinologic disorders* are responsible for fewer than 5% of instances of ED. The etiologic significance of the hypothalamic–pituitary–testicular axis in ED is unclear. Their effect on libido and sexual behavior is well established, but the effect of androgens on normal erectile physiology is poorly understood. It has been proved that testosterone enhances sexual interest, increases the frequency of sexual acts, and increases the frequency of nocturnal erections but has little or no effect on fantasy-induced or visually stimulated erections [2].
  - Hypogonadotropic hypogonadism is rare, its main charachteristic beeing the delayed puberty;
  - Hypergonadotropic hypogonadism (Klinefelter's syndrome, surgical orchiectomy) may decrease libido while potency may persist.
  - Late onset hypogonadism may lead to ED by decreasing the hormonal levels in a patient who previously had a normal androgenic function.
  - Hyperprolactinemia (pituitary adenoma, craniopharyngioma, drugs) is associated with low or low– normal levels of serum testosterone, its effects on erectile function appear to be centrally mediated. Hyperthyroidism is commonly associated with diminished libido and, less frequently with ED, while ED associated with hypothyroid states has been reported and may be secondary to associated low levels of testosterone secretion and elevated levels of prolactin.
- *Renal failure*: Approximately 50% of dialysis–dependent uremic patients suffer from ED, but improvement after transplantation occurs in many patients mostly because of reversal of the anemia associated with chronic renal failure and improvement in uremic neuropathy. In this case, the psychogenic etiology cannot be neglected
- *Neurogenic* It is estimated that 10–19% of the organic ED are neurogenic [3]. The main causes are:
  - intracerebral (Parkinson's Disease, cerebrovascular disease–especially efferent pathways from the medial preoptic area may be affected in addition to higher cortical functions, affecting sexual response; other causes are: stroke, encephalitis, or temporal lobe epilepsy)
  - spinal cord (trauma – psychogenic erections are not possible in patients with complete lesions above T12; up to 75% of patients with multiple sclerosis have sexual disfunction; myelodysplasia).
  - peripheral nerves are also affected in alcoholic neuropathy, diabetic neuropathy (most common cause), after surgery (radical pelvic surgery) and trauma.
- *Trauma*: pelvic fractures with ruptured posterior urethra. The damage to the neurovascular bundle or to the internal pudendal or common penile artery at the time of injury is predominantly responsible for most of the ED seen following these injuries. Perineal trauma, considered as a ‘hidden’ cause of ED, is often considered to be psychogenic, but neurovascular lesions may occur. Bicycle accidents and extensive bicycle riding account for a significant portion of these blunt perineal injuries, most of them during childhood.
- *Penile diseases*: vascular lesions in priapism, Peyronie's disease or other traumatic lesions of tunica albuginea or congenital deformities, can cause ED.
- *Malignant diseases*: lower abdomen or pelvic organs malignancies may cause organic ED. However, in the vast majority of malignancies, the psychogenic etyology is considered the most important
- *Iatrogenic*: consists in aortic or peripheral vascular surgery, renal transplantation (especially if a second contralateral transplantation is performed with end–to–end hypogastric artery anastomosis), perineal irradiaton (leads to fibrosis of cavernosal erectile tissue), cavernosal spongiosal shunts performed for the emergency treatment of priapism, abdominal perineal resection of the rectum, radical prostatectomy or cystoprostatectomy (the incidence of ED can be lowered to 40%–60% if nerve sparring techniques are used), transurethral sphincterotomy (should avoid incision at the 3 o'clock and 9 o'clock positions to prevent thermal injury to the cavernosal arteries). Other procedures may cause psychogenic ED.

*Drugs*: Medications inducing impotence can constitute up to 25% [4] of all cases. Patients taking medications affecting the autonomic nervous system or cardiovascular system may benefit from change or modify these medications, but attention must be paid to the risks involved in this changes.(Table 2)

**Table 2:** Various medications associated with ED

Centrally acting agents	Anticholinergic agents	Hyperprolactinemic agents	Sympatholytic agents	Antiandrogenic mechanism
Marijuana	Antimuscarinic	Estrogens	Bretylium	Spironolactones
Reserpine	Antihistamines	Phenotiazines	Reserpine	Estrogenes
Chlonidine	Chlonidine	Haloperidol	Clonidine	Cyproterone
Phenotiazines	antidepressants	Metoclopramide	Guanetidine	acetate
Ethanol	Phenotiazines	Imipramine	Adrenergic	Dysopiramide
Opioids		Reserpine	blockers	Ketoconazole
Methyldopa		Opiates	Methyldopa	Cimetidine
		Methyldopa		

## Treatment

Nowadays, the treatment for ED offers many more or less invasive options. If the diagnostic algorithm diagnosed a primary cause, the treatment will aim to eliminate the cause, hoping that ED will disappear subsequently. Despite the continual evolution of medicine, in most cases specific therapy for ED is still necessary, ranging from minor life–style changes to complex vascular surgery.

**Non surgical therapy** includes:

1. **Psychological therapy**, especially sex therapy is recommended for the patients with evidence of psychogenic ED and no detectable organic cause. A short course (4 to 12 weeks) of sex therapy should be prescribed. Family planning may also help fighting ED, if the cause is somehow interconnected [ 5].
2. **Lifestyle changes**, when ED is linked with obesity, initial stages of diabetes mellitus, etc. There is some evidence that ED may spontaneously subside if the general health status of the patient improves.
3. **Medication Change** – if ED is caused by medications.
4. **Herbal or vitamin supplements** are being used for centuries in treating ED, and they still have some role. Although some studies suggest that there is a high rate of placebo responders, herbal supplements are clinically proved to improve sexual function [6].
5. **Pelvic floor** exercises may reduce ED, although there is only limited evidence supporting that theory [7].
6. **Hormonal therapy**, more specific testosterone replacement, is proved as very effective if the cause of ED is the low level of testosterone. Some authors suggest that the testosterone level should be checked in all patients presenting for ED [8]. However, if low testosterone is diagnosed, further testing is necessary to rule out a metabolic syndrome. Nowadays, testosterone is available in several presentation, including patches, gels, pellet, oral pills, and buccal agents. The literature suggests that the results are similar, regardless the way of administration. The only major issue seems to be the correct indication for testosterone suppression.
  Recent evolutions developed a preparation of *dihydrotestosterone*, promising better results in hypogonadal men with a propensity to gynecomastia or boys with constitutionally delayed puberty [9].
  *Dehydroepiandrosterone* has a controversial role in improving the treatment of ED, although some data may suggest improvement of sexual function in treated men [10].
  *Human Chorionic Gonadotropin* proved effective when administered to aged men with testosterone levels in the lower range of normal. The results included a decrease in fat mass, an increase in body mass and no effect on muscle strength [11].
7. **Oral therapy** – phosphodiesterase type–5 inhibitors (PDE–5)
  Since 1998, when **sildenafil citrate (Viagra)** was introduced, the management of men with ED has revolutionized. Sildenafil increases the effects of the blood pressure lowering medications of nitrates, e.g. isosorbide dinitrate (Isordil), isosorbide mononitrate (Imdur, Ismo, Monoket), nitroglycerin (Nitro–Dur, Transderm–Nitro) that are used primarily for treating angina. Sildenafil increased the number of patients using other therapeutic modalities such as intracavernosal injections and penile prostheses. The drug blocks the hydrolysis of cyclic GMP, enhancing the accumulation of cyclic GMP and potentiating the relaxant effects of nitric oxide. Sildenafil is effective in treating ED resulting from a variety of organic causes, including diabetes mellitus. Sildenafil is contraindicated in patients who require nitroglycerine to treat myocardial ischemia. Since then, new PDE–5 inhibiters were developed, each promising to be faster, better or longer acting than the original.
  **Tadalafil (Cialis)** is also a new, potent and selective PDE5 inhibitor, it's most unique and identifying characteristic being its long half–life of 17.5 hours, compared with 4 hours for sildenafil (Viagra) and vardenafil (Levitra) [12]. Tadalafil is a safe, well–tolerated, and efficient treatment for all severities and etiologies of ED, and its long half–life lends itself to a longer therapeutic window with on–demand dosing and effective steady–state plasma concentrations with once– daily dosing.
  Tadalafil may also improve lower urinary tract symptoms in men with benign prostatic hyperplasia (BPH). Researchers from Nashville, Dallas, San Antonio and Indianapolis presented these findings during the 104^th^ Annual Scientific Meeting of the American Urological Association (AUA). In this study [13], researchers randomly separated 200 men, with an age equal to or older than 40 years and at least a six month diagnosis of BPH-LUTS with an International Prostate Symptom Score (IPPS) greater than or equal to 13, into two groups taking either 20 mg of tadalafil once daily or a placebo. After 12 weeks of treatment, the men taking tadalafil experienced improved detrusor pressure at urinary flow rate, peak flow rate (Qmax), bladder capacity, post–void residual volume and bladder voiding efficiency. Relative symptom improvement in the IPSS also was significantly better in the tadalafil group. At the end of the study, the proportion of obstructed patients in the placebo group increased, while the proportion in the tadalafil group decreased [14].
  **Vardenafil (Levitra)** is a potent and selective PDE5 inhibitor. Data showed [15] that 38.9% preferred vardenafil compared to 34.5% sildenafil (26.6% had no preference). Vardenafil was significantly superior to sildenafil in terms of erectile function, intercourse satisfaction and overall satisfaction. There were also a significant higher percentage of positive responses for vardenafil with regards to erection hardness for penetration, maintenance of erection, maintenance until completion, and erection confidence.
  Long–term use of Vardenafil (Levitra) at the maximum recommended dosage does not adversely affect sperm concentration, total sperm count per ejaculate, or sperm morphology or motility, a study recently found [16]. Because recent data suggest that many men in their reproductive years now use phosphodiesterase–5 (PDE–5) inhibitors for erectile dysfunction (ED), Keith Jarvi, professor of surgery at the University of Toronto and his colleagues examined the long– term effects of these agents on sperm concentration and other semen characteristics. They compared the effects of vardenafil (20 mg), sildenafil (Viagra, at 100 mg), and placebo in 200 men aged 25–64 years (mean age 39 years). Some men did not have ED and the others had ED but were able to produce semen samples without ED therapy. Following an unmedicated screening period of four weeks, each subject underwent daily treatment with vardenafil, sildenafil, or placebo for six months. After the treatment phase, men with abnormal semen analyses also participated in a three– month follow–up. The primary variable was the proportion of subjects treated with vardenafil who had a 50% or greater reduction in mean sperm concentration from baseline to six– month last observation carried forward (LOCF), compared with placebo–treated men. An analysis of the mean changes from baseline to six–month LOCF revealed that sperm concentration was 2 million/mL for vardenafil, 8 million/mL for sildenafil, and 1 million/mL for placebo. The percentage change of normal sperm morphology was–1% for vardenafil, –1% for sildenafil, and 0% for placebo. The change in total sperm motility was similar for vardenafil, sildenafil, and placebo. The researchers observed no statistically significant difference in the median changes in total sperm count per ejaculate (millions) for men taking vardenafil, sildenafil, or placebo.
  The current recommendations in medical treatment of ED is that all three PDE–5 inhibitors should be tried (one at a time), before shifting to more invasive therapy [15].
8. **Intracavernous injection**– although long known and used since the initial demonstration of Brindley in 1983, it is in constant evolution and development. Most commonly used are papaverine (alkaloid isolated from opium), phemtolamine and alprostadil (widely known as Caverject). Some authors report good results when using combination of two or three agents [16].
9. **Intraurethral Therapy** proved effective in treating ED during the last decades. The MUSE device, containing alprostadil, is the only FDA approved treatment.
10. **Vacuum Erection Device (VED)** is one of the most common choices of noninvasive therapy for ED. It consists of a cylindrical component and a suction device that the patient places around the penis to create negative pressure and achieve an erection. Maintenance of erection is then accomplished with an elastic constriction ring placed at the base of the penis. Patients with significant peripheral vascular disease, those receiving anticoagulants, and diabetics are generally not good candidates for the VED. Patient acceptance and satisfaction with VED in all types of ED (including diabetic ED), have been reported to be 68% to 83%, while the most frequent complications remain difficulty with ejaculation, penile pain, ecchymoses, hematomas, and petechiae (patients taking aspirin or warfarin are more likely to develop complications related to vascular fragility). Vacuum erection devices (VED), because of their ability to draw blood into the penis regardless of nerve disturbance, have become the centerpiece of penile rehabilitation protocols. Even nerve–sparing radical prostatectomy damages the cavernous nerves and leads to temporary erectile dysfunction (ED) in men recovering from prostate cancer surgery. Historically, patients recovering from prostate cancer surgery have been advised that the return of erectile function (EF) can take from 6 to 18 months, or even longer. Recently, there has been a growing movement to proactively treat patients postoperatively for presumed nerve damage to stimulate nerve recovery and possibly reduce the degree of irreversible damage [17].

**Surgical techniques** used in the treatment of ED aim to restore erection by means of intracavernous prosthesis or to cure other cause that led to ED.

1. **Penile prostheses** are represented by several constructive models: semirigid rod, positionable, two piece inflatable or three piece inflatable. Although the cost and high invasiveness of the procedure may reduce its expansion in the general population, the results are generally better, in terms of errection, personal and partner satisfaction. Future evolutions will most likely try to improve the long term mechanical reliability [18].
2. **Vascular surgery** for ED became widespread at the  end of the 1980s, but the poor long term results have somehow compromised the initial enthusiasm. The American Urological Association Guidelines still considers this type of intervention as experimental, due to the lack of consistent data and standardized procedures [19]. The main procedures used today are penile revascularization and penile venous surgery, recommended only for selected patients and offering fair long– term results

## Future developments

Erectile dysfunction grew, in less than two decades, from a border–line disease approached by some specialists, to a specialty by itself, a multi–billion dollar business or a field of continuous research. The evolution is far from being over, and many new spectacular evolutions are more than likely. From minor improvements in current treatments to breakthrough new therapies, erectile dysfunction has still many to offer [20].

An innovative drug–delivery system known as **nanoparticles** encapsulating nitric oxide and prescription drugs looks promising for topical treatment of ED, according to a new study by scientists at Albert Einstein College of Medicine of Yeshiva University [21]. The new system, tested successfully on a small number of animals, could potentially prevent side effects associated with oral ED medications, if study results can be replicated in humans. That could mean safer and more effective ED therapy for millions of men with heart disease and other health problems affecting erectile function. The study is published recently in the online edition of the *Journal of Sexual Medicine, September 2009* [21].

The drug–delivery system, developed by Einstein scientists, consists of nanoparticles, each smaller than a grain of pollen that can carry tiny payloads of various drugs or other medically useful substances and release them in a controlled and sustained manner. An effective topical therapy could be especially significant for those ED patients particularly men with diabetes who have reduced levels of nitric oxide (NO), the signaling molecule that dilates blood vessels responsible for erectile activity. These men, who often aren't helped by oral PDE5 inhibitor drugs, may benefit from direct application of NO or the PDE5 inhibitors. Five of the seven rats treated with the NO–containing nanoparticles, and all 11 rats treated with nanoparticles encapsulating NO plus sialorphin or tadalafil showed significantly improved erectile function. None of the seven rats in a control group, which received empty nanoparticles, showed any improvement. ‘The response time to the nanoparticles was very short, just a few minutes, which is basically what people want in an ED medication,’ adds Dr. Davies. ‘In both rats and humans, it can take 30 minutes to one hour for oral ED medications to take effect’

VIVUS, a pharmaceutical company dedicated to the development and commercialization of novel therapeutic products, today announced it has initiated a second pivotal Phase 3 clinical trial of **avanafil **, its investigational new drug for the treatment of erectile dysfunction (ED). Avanafil is a next– generation, fast–acting, selective, investigational oral phosphodiesterase type 5 inhibitor. The study, REVIVE– Diabetes (TA–302), is a multicenter, randomized, double–blind, placebo–controlled trial and will evaluate the safety and efficacy of avanafil in the treatment of ED in men with type 1 or type 2 diabetes. Subjects who meet the inclusion criteria will undergo a four–week non– treatment run–in period followed by 12 weeks of treatment. The co–primary endpoints of the study will be the improvement in the erectile function as measured by changes in the sexual encounter profile (SEP) questions 2 and 3, and improvement in erectile function as measured by the erectile function domain score of the International Index of Erectile Function (IIEF) [22]. The SEP is a self–administered patient diary and the IIEF is a patient questionnaire; both are used as standard diagnostic tools to assess erectile dysfunction. REVIVE–Diabetes is the second of three planned pivotal studies in the avanafil Phase 3 development program.

**ZoraxelTM** is being developed as an orally administered, on–demand tablet. It has a well established and excellent safety record in humans and appears to lack severe side effects associated with standard of care PDE–5 inhibitor ED drugs, such as priapism, severe hypotension, myocardial infarction, sudden death, increased intraocular pressure and sudden hearing loss. ZoraxelTM is a centrally acting, dual enhancer of neurotransmitters in the brain, whereas PDE-5 inhibitors only target end organ erectile function and work in peripheral blood vessels. In preclinical animal models, ZoraxelTM has significantly improved all three functions of sexual activity, i.e. sexual arousal, erection, and release, and may be a more effective ED treatment for patients who are responsive or unresponsive to PDE–5 inhibitors. The Zoraxel Phase 2a trial is a double blind, placebo–controlled, dose ranging study conducted at three U.S. study sites in up to 40 male subjects ages 18 to 65 with ED for six months. Main study endpoints for the 8–week treatment period were the Sexual Encounter Profile (SEP) and the International Index of Erectile Function (2EF), both of which are validated surveys for assessing erectile function. Planning is underway for initiation of Phase 2b clinical studies.

**Momentary Squeeze (MS) Pump (by AMS)** was first introduced nearly two years ago, and Dr. Eid has implanted it with great success in patients suited to the CX–cylinder and LGX–cylinder versions. (The LGX model offers the potential for increased penile length in patients complaining of penile shortening after radical prostatectomy.) Both cylinders now feature redesigned proximal ends. At only 9.5mm in diameter, the ends allow for significantly easier insertion for patients with fibrotic proximal corporal bodies.

The MS Pump's advantages include a smaller profile (which enables a more discreet placement) and one–touch deflation. However, this quality can result in operational difficulties with inflation (more difficult to get a hold of within the scrotum) and deflation (small button can initially be difficult to find). In addition, its new lockout valve has the advantage of preventing auto–inflation (potentially embarrassing and/or painful), but can at times make it difficult to initiate inflation. For these reasons, Dr. Eid recommends this pump for the younger patient with an average–sized penis.

**Tactile Pump (700 CX Series by AMS** ‘The AMS 700 CX Series with the Tactile Pump remains my prosthesis of choice for the older patient where pump concealment is not an issue,’ says Dr. Eid. ‘This pump is large, easily palpable, remains the softest to inflate and has a very large deflation footprint, which is quickly recognized by the patient.’ 

**Titan Pump – Coloplast**. The FDA approved the improvements to the pump component of the Coloplast Titan prosthesis in July 2008. Although the cylinders and reservoir remain the same as the previous model, the pump now features a one–touch– release (OTR) deflation valve – easy for patients to locate and operable with one hand. In addition, the pump offers a non–bulky, low–profile size; enhanced silicone for higher threshold ‘tear strength’ (likely to result in increased product durability, an issue with previous versions); and an overall simplicity likely to decrease repeat office visits, phone calls, and repeat training time. From a hospital standpoint, intraoperative prep of the device provides for easier priming of the implant system (the removal of excess air prior to filling) and may reduce slightly the overall intraoperative time.

In a recent study (‘Evaluation of Three Penile Prosthesis Pump Designs in a Blinded Survey of Practitioners,’ Urologic Nursing, 2008), 32 medical professionals, all familiar in teaching the operation of penile implants to patients, reviewed currently available penile pumps. The blindfolded reviewers, examining the pumps under time constraints through mock scrotal sacs, were asked to rate device:

- ease of location of deflation valve
- ease of inflation
- ease of deflation
- anticipated ability to train patients in clinical setting.

The well known PDE–5 inhibitors may soon live a second youth, after new *talents* of their molecules are being revealed. Sildenafil citrate, more widely known as Viagra, has already been shown to improve heart function and may one day have value in either treating or preventing heart damage due to chronic high blood pressure [23]. The key, investigators say, is sildenafil's effects on a single protein, RGS2, newly identified in the latest study as an essential link in the chain reactions that initially protect the body's main blood–pumping organ from spiraling into heart failure. Experimenting in mice, the team of heart experts first established that after a week of induced high blood pressure, the hearts of animals engineered to lack RGS2, or regulator of G–protein signaling 2, quickly expanded in weight by 90 percent. Almost half the mice died of heart failure. In mice with RGS2, by contrast, the dangerous muscle expansion, known as hypertrophy, was delayed, growing only 30 percent, and no mice died [24]. Subsequent tests treating hypertensive mice that had RGS2 with sildenafil showed enhanced buffering, with less hypertrophy, stronger heart muscle contraction and relaxation, and as much as 10 times lower stress–related enzyme activity compared to their untreated counterparts. In mice lacking RGS2, sildenafil had no effect [25].

**Centraly acting drugs**, such as yohimbine, trazodone or apomorphine may replace at some point the PDE–5 inhibitors. *Yohimbine hydrochloride* is an adrenergic antagonist, acting centrally; its effect is to ' increase cholinergic and decrease adrenergic activity. Yohimbine also acts as a mood stimulant. Its efficacy rate is only about 20% to 25% overall, and it seems to be most effective in patients with psychogenic ED. Trazodone, already known for its antidepressant effect, showed some effect in improving erections, despite several side effects. **Apomorphine** existed for a while on the market as ED therapy, but some issues concerning the way of administration limited very much its success. Future evolutions will most likely overcome this downside, putting in a new light the centrally acting drugs [26].

**Melanocortin Receptor Agonists** are used is controlling food intake and energy expenditure, but also proved promising in modulating erectile function and sexual behavior [27].

The end of 2006 was marked by the first clinical trial on humans, using gene therapy for treating ED. The results suggest that gene therapy lasts for months, eliminating the needs for on– demand therapy, opening the door towards the future of treatment for ED. The authors of the study are making several comments regarding possible complications and side effects of gene therapy, but also challenging results and possible ways of development [26].
